# Internet-Based Inquiries From Users With the Intention to Overdose With Over-the-Counter Drugs: Qualitative Analysis of Yahoo! Chiebukuro

**DOI:** 10.2196/45021

**Published:** 2023-11-22

**Authors:** Azusa Kariya, Hiroshi Okada, Shota Suzuki, Satoshi Dote, Yoshitaka Nishikawa, Kazuo Araki, Yoshimitsu Takahashi, Takeo Nakayama

**Affiliations:** 1 Department of Health Informatics, Graduate School of Medicine & School of Public Health Kyoto University Kyoto Japan; 2 Department of Social ＆ Community Pharmacy, School of Pharmaceutical Sciences Wakayama Medical University Wakayama Japan; 3 Institute for Clinical and Translational Science Nara Medical University Hospital Kashihara Japan; 4 Department of Pharmacy Kyoto-Katsura Hospital Kyoto Japan

**Keywords:** abuse, consumer-generated media, CGM, overdose, over-the-counter drug, OTC drug, question and answer site, Q and A site

## Abstract

**Background:**

Public concern with regard to over-the-counter (OTC) drug abuse is growing rapidly across countries. OTC drug abuse has serious effects on the mind and body, such as poisoning symptoms, and often requires specialized treatments. In contrast, there is concern about people who potentially abuse OTC drugs whose symptoms are not serious enough to consult medical institutions or drug addiction rehabilitation centers yet are at high risk of becoming drug dependent in the future.

**Objective:**

Consumer-generated media (CGM), which allows users to disseminate information, is being used by people who abuse (and those who are trying to abuse) OTC drugs to obtain information about OTC drug abuse. This study aims to analyze the content of CGM to explore the questions of people who potentially abuse OTC drugs.

**Methods:**

The subject of this research was *Yahoo! Chiebukuro*, the largest question and answer website in Japan. A search was performed using the names of drugs commonly used in OTC drug abuse and the keywords *overdose* and *OD*, and the number of questions posted on the content of OTC drug abuse was counted. Furthermore, a thematic analysis was conducted by extracting text data on the most abused antitussive and expectorant drug, *BRON*.

**Results:**

The number of questions about the content of overdose medications containing the keyword *BRON* has increased sharply as compared with other product names. Furthermore, 467 items of question data that met the eligibility criteria were obtained from 528 items of text data on BRON; 26 codes, 6 categories, and 3 themes were generated from the 578 questions contained in these items. Questions were asked about the effects they would gain from abusing OTC drugs and the information they needed to obtain the effects they sought, as well as about the effects of abuse on their bodies. Moreover, there were questions on how to stop abusing and what is needed when seeking help from a health care provider if they become dependent. It has become clear that people who abuse OTC drugs have difficulty in consulting face-to-face with others, and CGM is used as a means to obtain the necessary information anonymously.

**Conclusions:**

On CGM, people who abused or tried to abuse OTC drugs were asking questions about their abuse expectations and anxieties. In addition, when they became dependent, they sought advice to quit their abuse. CGM was used to exchange information about OTC drug abuse, and many questions on anxieties and hesitations were posted. This study suggests that it is necessary to produce and disseminate information on OTC drug abuse, considering the situation of those who abuse or are willing to abuse OTC drugs. Support from pharmacies and drugstores would also be essential to reduce opportunities for OTC drug abuse.

## Introduction

### Background

In recent years, public concern with regard to drug abuse has grown rapidly across countries. In the United States, heroin and opioid abuse is a significant public health concern [1]. Over-the-counter (OTC) drugs such as dextromethorphan and loperamide are also a problem in drug abuse [2]. A previous systematic review that focused on OTC drug abuse described its pharmacological effects: dextromethorphan, acting as an N-methyl-D-aspartate receptor antagonist, causes hallucinogenic and dissociative states; potent H1 antihistamines with anticholinergic properties cause sedative, anxiolytic, and euphoric states; and codeine (opioid), acting as a selective agonist of the μ opioid receptor, causes euphoric, elate, and hallucinogenic states [3]. In Japan, drugs containing ingredients such as dihydrocodeine phosphate are known to have potential for abuse. One of the opportunities to make this known was the report on the *Survey of Drug-Related Psychiatric Disorders in Psychiatric Facilities in Japan* [4], a shared research project granted in aid for scientific research by the Ministry of Health, Labour and Welfare in the fiscal year (FY) 2008 (April 2008 to March 2009). The report revealed that people who abuse OTC drugs accounted for approximately 40% of teenaged patients with drug-related disorders [4]. This survey was conducted over time, showing a sharp increase in the number of teenaged patients with drug-related disorders owing to OTC drugs between FY 2014 and 2018 [4]. In Japan, OTC drugs are different from illegal drugs such as methamphetamine because they are inexpensive and easily available to anyone and thus pose different difficulties in combating them. Unlike many other countries, OTC drug abuse is more likely to be a problem in Japan because drugs containing dihydrocodeine phosphate are generally available as OTC drugs [5].

The potential problem with OTC drugs is that they are “easy to abuse,” but their abuse has serious mental and physical effects, including poisoning symptom and dependence, and in many cases, hallucinations, euphoria, dissociative states, and even death that require specialized treatment [3,6-9]. As a matter of fact, the existence of people who potentially abuse OTC drugs who do not show enough symptoms to visit a mental hospital or drug addiction rehabilitation center [10] but are at high risk of falling into dependence in the future is considered a serious problem [11].

Nowadays, social media, such as question and answer (Q&A) websites (eg, *Yahoo! Chiebukuro*, the Japanese counterpart of *Yahoo! Answers*) and X (formerly known as Twitter), is often used on the internet, where users can transmit information themselves [12-14]. People who abuse (and attempt to abuse; the same applies hereafter) OTC drugs also use social media as a medium to quickly obtain the information they seek without being identified [15-20]. Social media that generate content through postings by ordinary users mainly through the internet is called *consumer-generated media (CGM)* or *user-generated content* [21,22]. Registered users, that is, those with a user ID, anonymously post questions on the Q&A website, one of the knowledge communities. Subsequently, knowledge is shared by another user posting an answer and so on [23]. As diverse information on drugs is exchanged on Q&A websites, it is indispensable to analyze the use of Q&A websites by people who potentially abuse OTC drugs to obtain information on the actual status of OTC drug abuse from the perspective of the persons concerned.

In Japan, studies on people who abuse OTC drugs in medical institutions [4,6] and studies on sales methods of OTC drugs at risk for abuse [11,24,25] have been reported. However, there have so far been no studies on people who potentially abuse drugs who have not visited a specialized institution. In other words, the currently revealed reality of OTC drug abuse is only the “tip of an iceberg” [26].

### Objectives

This study aimed to analyze questions about OTC drug abuse on a well-known Q&A website, thus exploring the questions that people who potentially abuse OTC drugs were carrying.

## Methods

### Data Source and Search Keywords

Considering the current situation of social media on OTC drug abuse as mentioned earlier, we used data on questions posted on Yahoo! Chiebukuro, the largest Q&A website in Japan, which started its posting service in April 2004 [27]. It is a user-participatory knowledge community in which registered users ask and answer each other’s questions [28]. Anyone can view posted questions and answers even if they are not registered as a user. In this study, only questions posted on Yahoo! Chiebukuro were included in the analysis. In the abuse of OTC and prescription drugs, there are cases where the abuse is the result of seeking symptom relief from the original effects of the drug and cases where the overdose is intentionally taken for self-harm or suicide purposes or for *distracting anxiety* or *increasing motivation* rather than for the original effects of the drug [29]. The rapid increase in the latter is particularly problematic. The term commonly used on the web to refer to overdoses is *overdose* or *OD* (abbreviation for overdose). Overdose and OD, rather than *KARYO-FUKUYAKU* (overdose) and *RAN’YO* (abuse), were used to talk about the content of OTC drug abuse. Therefore, the words overdose and OD were widely used as keywords in the search for the purpose of extracting the content of abuse in this study. In addition, as we confirmed that cryptic words (eg, *KIN-PAB* for *PABRON GOLD*, a multi-ingredient cold medication, and *RETASU* for *RESTAMIN*, an antiallergic agent) were frequently used on the internet regarding overdose, we added them to the search terms. The categories within Yahoo! Chiebukuro were not considered during the search.

### Aggregating Data on the Number of Questions

Several product names were extracted from the product names of OTC drugs used for purposes other than their original purpose as indicated in a previous survey [4]. With reference to previous studies, a selection was made within the researchers regarding OTC drugs with a risk of abuse (Table 1). Questions were then extracted by searching with those product names and the keywords *overdose* and *OD* on the Yahoo! Chiebukuro website. The search was conducted on October 27 and November 17 to 20, 2019. We chose the 8 drugs with the highest number of question postings on overdose over the 15-year period from FY 2004 to 2018, the period covered by the study. In addition, we read the text of the extracted questions, summarized the number of questions that included OTC drug abuse in the content, and presented a graph showing the trends in question postings over time. We also counted the number of questions per product for each 5-year period and listed the names of the top 5 products in a table.

**Table 1:** Number of cases of over-the-counter abuse from previous studies [4] and the number of questions in Yahoo! Chiebukuro.

Product names	Cases, n	Questions, n
*BRON* ^a^	158	522
*PABRON* ^a^	34	181
*WUTT* ^a^	32	47
NARON	16	16
*EVE* ^a^	15	100
DREWELL	12	18
*BUFFERIN* ^a^	12	132
CONTAC	10	38
TONIN	10	12
SEDES	6	10
BENZA	6	18
*RESTAMIN* ^a^	6	139
*LOXONIN* ^a^	6	80
LULU	5	27
*ESTARON* *MOCHA* ^a^	4	25
RISURON	4	0
PA or PL	3	1
NORSHIN	3	6
KAIGEN	2	0
KERORIN	2	0
PRECOL	2	2

^a^The 8 drugs extracted in this study.

### Analyzing Question Content: Thematic Analysis

#### Data Extraction

Among the OTC drugs used for purposes other than their original purpose, *BRON*, a compound antitussive and expectorant drug, was the most commonly used [4]. Therefore, data were extracted by searching for the keywords *BRON overdose* and *BRON OD* on Yahoo! Chiebukuro. Data were searched and displayed by entering keywords on the Yahoo! Chiebukuro website. We copied each question displayed and attached it to an Excel file (Microsoft Corporation). The data were then compiled into a list to create textual data. We removed parts of the extracted preprocessed question data that were not required for the analysis. We then reconstructed the questions with contents that could be the subject of this study. Simultaneously, if a single question contained multiple questions, each question was analyzed as a separate code.

#### Analysis

We performed a basic thematic analysis [30,31]. The thematic analysis proposed by Braun and Clarke [32] was used as a reference. We extracted only the content of the questions from Yahoo! Chiebukuro postings, excluding background information such as age, history of mental illness, and family environment to inductively create codes, categories, and themes. We conducted researcher triangulation with 3 pharmacists and 1 physician to maintain analytical certainty during the analytical process. We used NVivo 12 Plus (Lumivero) for Windows as a qualitative data analysis software. This study was conducted in accordance with the guidelines for publishing qualitative research [33].

### Ethical Considerations

This study did not involve human participants at the individual level. Further, the data used in this study were open data and anonymous information. Therefore, they are not subject to ethical guidelines.

## Results

### Data on Number of Questions

**Figure 1:**
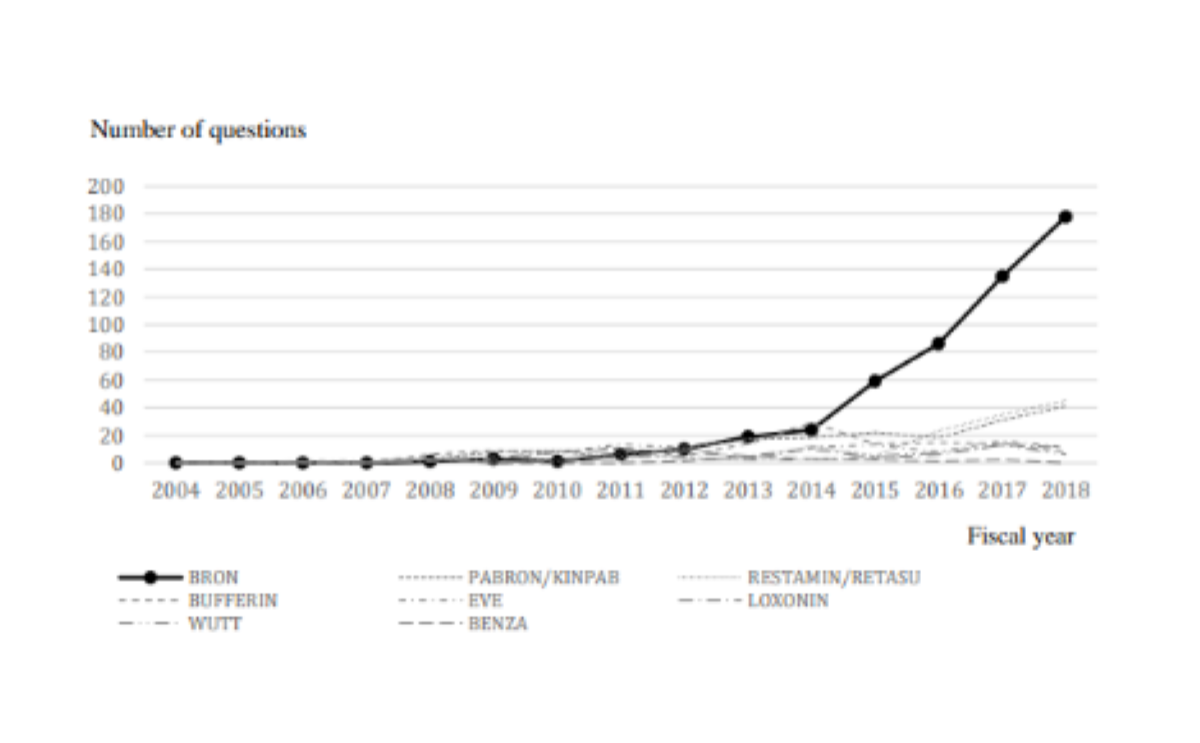
Number of questions about the content of overdoses.

The 8 drugs with the highest number of question postings on overdose in the 15-year period from FY 2004 to FY 2018 are shown in Table 2. The number of questions about overdose containing the keyword *BRON*, a compound antitussive and expectorant drug, increased sharply compared to other products (Figure 1). Table 3 shows the top 5 products with the highest total number of cases for each 5-year period. The number of questions about overdose that included the keyword *BRON* increased and replaced the analgesic *BUFFERIN* and the multi-ingredient cold medication *PABRON* in terms of the number of questions.

**Table 2:** Pharmaceutical ingredients.

Product name	Japanese name	Ingredients of potential abuse
BENZA	ベンザ	Dihydrocodeine phosphate, pseudoephedrine hydrochloride, and anhydrous caffeine
BRON	ブロン	Dihydrocodeine phosphate, DL-methylephedrine hydrochloride, and anhydrous caffeine
BUFFERIN	バファリン	Aspirin (acetylsalicylic acid)
EVE	イブ	Allylisopropylacetylurea and anhydrous caffeine
LOXONIN	ロキソニン	Allylisopropylacetylurea and anhydrous caffeine
PABRON or KIN-PAB	パブロンまたは金パブ	Dihydrocodeine phosphate, DL-methylephedrine hydrochloride, acetaminophen, and anhydrous caffeine
RESTAMIN or RETASU	レスタミンまたはレタス	Diphenhydramine hydrochloride
WUTT	ウット	Bromovalerylurea and allylisopropylacetylurea

**Table 3:** Number of questions about overdose—top 5 products with the highest total number of cases for each 5-year period.

Rank	Fiscal year 2004-2008			Fiscal year 2009-2013			Fiscal year 2014-2018	
	Product name	Questions, n	Product name		Questions, n	Product name		Questions, n
1	BUFFERIN	6	PABRON or KIN-PAB		48	BRON		483
2	EVE	5	BUFFERIN		46	PABRON or KIN-PAB		130
3	BENZA	4	BRON		39	RESTAMIN or RETASU		120
4	PABRON or KIN-PAB	3	EVE		36	BUFFERIN		80
5	RESTAMIN or RETASU and WUTT	2	LOXONIN		35	EVE		59

### Question Analysis

**Figure 2:**
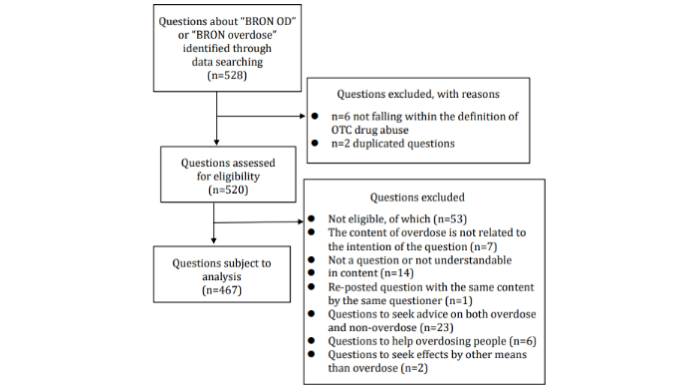
Data extraction flow diagram. OD: overdose; OTC: over-the-counter.

From November 25 to December 6, 2019, we searched the Yahoo! Chiebukuro website for the keywords *BRON OD* and *BRON overdose* and extracted 528 questions. Among them, we excluded 6 questions not relating to OTC drug abuse and 2 duplicate questions, which resulted in 520 questions subject to our analysis. From these, we excluded 53 questions that met the exclusion criteria established in consultation with our collaborators after reviewing the question contents, leaving 467 questions for analysis in this study (Figure 2). If a single question contained more than 1 piece of content, we assigned a code for each piece of content. The number of questions analyzed in this study was 578, which was greater than the number of questions asked.

We established 26 codes, 6 categories, and 3 themes from the 578 questions (Table 4). The codes are numbered and italicized in the text, and some of the actual questions are cited below in *italics*. The actual postings cited in the text use the same numbering as the codes to tie each of them to its related code. In doing so, the actual postings with their respective codes are distinguished from the codes themselves by being cited in *italics*.

**Table 4:** Code table.

Theme and category		Code
**1. Expectations for overdose**		
	1. Information necessary to achieve the effect of overdose sought by drug users	1. Type and dosage of drug to achieve the desired effect of overdose 2. How to obtain drugs to be used for overdose 3. To know how to reach overdose 4. How long it takes to obtain the expected effect 5. Duration of the effect 6. Concerns about developing resistance to drugs
	2. Effects obtained from overdose	7. Questions about the effects of overdose 8. Questions about not feeling the effects they seek 9. Ways to feel more effects of overdose
**2. Anxieties about overdose**		
	3. Physical effects of overdose	10. Some effects that are not sought by overdose 11. Concerns about the duration of unwanted effects of overdose 12. Physical effects of the immediate postoverdose behaviors 13. Need to seek medical advice for overdose-related symptoms 14. Concerns about overdose in the long term 15. Dependence on overdose 16. Concerns about withdrawal symptoms from ongoing overdose 17. Bodily effects of the concomitant use of drugs and supplements intended for their original efficacy
	4. Effects of overdose on personal relationships	18. Anxiety about other people knowing about one’s own overdose 19. Anxiety about the results of the medical examination 20. Anxiety about the physician’s reaction during the visit
**3. Troubles in quitting overdose**		
	5. Questions about quitting overdose	21. Difficulty of quitting overdose 22. How to quit overdose 23. Alternative ways to quit overdose 24. Coping with mental distress when quitting overdose
	6. Questions about seeking help from the medical institution	25. Need to see a psychiatrist or psychosomatic physician 26. Hospital’s possible reaction when visiting a psychiatrist or psychosomatic physician

### Theme 1: Expectations for Overdose

#### Category 1: Information Necessary to Achieve the Effect of Overdose Sought by Drug Users

First, to overdose, it is necessary to obtain the drug to be overdosed with. Therefore, they asked questions about (1) *the type and dosage of drug to achieve the desired effect of overdose* and (2) *how to obtain drugs to be used for overdose*. After the drugs are in hand, the next step is (3) *to know how to reach overdose*. For this purpose, they asked about beverages for swallowing tablets, multiple types of drugs, combination with alcohol, time of day for overdose, time to be spent for taking drugs, frequency of overdose, etc. Most of them expected some kind of effect in overdose, asking (4) *how long it takes to obtain the expected effect* and (5) *the duration of the effect*. In addition, to ensure that the effects of overdose could be felt for a longer period, they asked about (6) *concerns about developing resistance to drugs*. Although most of the patients were looking for euphoria and fluffiness, some appeared to be looking for hallucination, hematemesis, and collapse:

This is the first time I try to overdose. How many tablets should I take for the first time?

Code 1

I would like to take OD. Can I buy BRON at a pharmacy or something? Will they check my age?

Code 2

Is it safe to take BRON and KIN-PAB together?

Code 3

How long does it take to feel fluffiness after taking BRON?

Code 4

How many hours does it take to lose its effect if I take 20 tablets of BRON OD?

Code 5

How can I make sure I don’t develop a resistance to BRON OD?

Code 6

#### Category 2: Effects Obtained From Overdose

They asked (7) *questions about the effects of overdose*, such as the kinds of effects obtained from overdose; (8) *questions about not feeling the effects they seek*; and (9) *ways to feel more effects of overdose*:

What happens mentally when one takes OD of BRON tablets? Does it make one feel better?

Code 7

I took 40 tablets of BRON and didn’t see any change. Is it normal?

Code 8

I took OD of BRON for the first time. I took 8 pills, ‘cause I thought I should double the dose. But it didn’t work at all. No nausea or anything. Only lightheadedness. How many pills should I take next time?

Code 9

### Theme 2: Anxieties About Overdose

#### Category 3: Physical Effects of Overdose

Some effects are sought by overdose and (10) *some effects are not sought by overdose*. There were cases where the patients asked about various symptoms such as hallucinations, nausea or vomiting, tremors or convulsions, effects on urination or defecation, eye effects, and itching, either because they were anxious beforehand or because they were distressed by the symptoms actually experienced. Thus, questions were asked about (11) *concerns about the duration of unwanted effects of overdose*; (12) *the physical effects of immediate postoverdose behaviors*, such as bathing; (13) *the need to seek medical advice for overdose-related symptoms*; and (14) *concerns about overdose in the long term*. In addition, there were questions on (15) *dependence on overdose* and (16) *concerns about withdrawal symptoms from ongoing overdose*. Other questions were asked about (17) *the bodily effects of the concomitant use of drugs and supplements intended for their original efficacy* such as psychiatric prescription drugs, low-dose pills, and supplements and whether the overdose of drugs would reduce the effectiveness of nonoverdose drugs taken:

I took 84 tablets of BRON and 35 tablets of gastrointestinal for OD. I'm shaking all over. Which hospital should I go to?

Code 10

I took 84 tablets of BRON about an hour ago...If I suffer and go to the hospital, will they wash my stomach?

Code 13

If I don’t take OD of BRON, my body gets lazy and won't move. Thoughts also become negative. I am worried if I don’t have it on hand. Is it possible to become so dependent on it after taking it only for a week or so?

Code 15

I have been taking low-dose pills (YAZ). If I take OD of BRON, will the contraceptive effect be diminished?

Code 17

#### Category 4: Effects of Overdose on Personal Relationships

On the basis of the perception that overdose is a bad thing, questions were asked about (18) *anxiety about other people knowing about one’s own overdose*, (19) *anxiety about the results of the medical examination*, and *(20) anxiety about the physician's reaction during the visit*:

Since a week or so, I have been taking OD of BRON, RESTAMIN, and painkillers every night...Should I talk to my family or boyfriend about my OD? I'm afraid I'll be scorned...

Code 18

Would a blood test reveal my OD of BRON?

Code 19

### Theme 3: Troubles in Quitting Overdose

#### Category 5: Questions About Quitting Overdose

Dependence through continuous leads to a situation where one cannot stop overdose even if they wanted to. There were questions on (21) *the difficulty of quitting overdose*, (22) *how to quit overdose*, (23) *alternative ways to quit overdose* that would have the same effect as overdose, and (24) *coping with mental distress when quitting overdose*:

I want to quit OD of BRON...I don’t feel like working without OD...What shall I do?

Code 22

It’s been a month since I quit OD of BRON. I did my best to stop but I want to take OD every day. Is there any way to control it?

Code 24

#### Category 6. Questions About Seeking Help From the Medical Institution

There was a question about (25) *the need to see a psychiatrist or psychosomatic physician*, although one had trouble stopping overdose. There was also a concern about (26) *the hospital’s possible reaction when visiting a psychiatrist or psychosomatic physician* because of overdose:

I can’t stop taking OD of BRON for cough medicine and OD of “SHIN-TONIN-EKI (new TONIN solution)”...I want to stop them. But I can’t stop. My face has changed so much that I can see it for myself. I am also experiencing physical discomfort. Should I consult with a psychosomatic physician? Can a psychosomatic physician in town help me if I say, “I’m taking OD of BRON”?

Code 25

I did not tell my past psychiatrist about my overdose. If I speak of my overdose habit, wouldn’t any drugs such as stabilizers or sleeping pills be prescribed?

Code 26

### Synthesis and Interpretation

**Figure 3:**
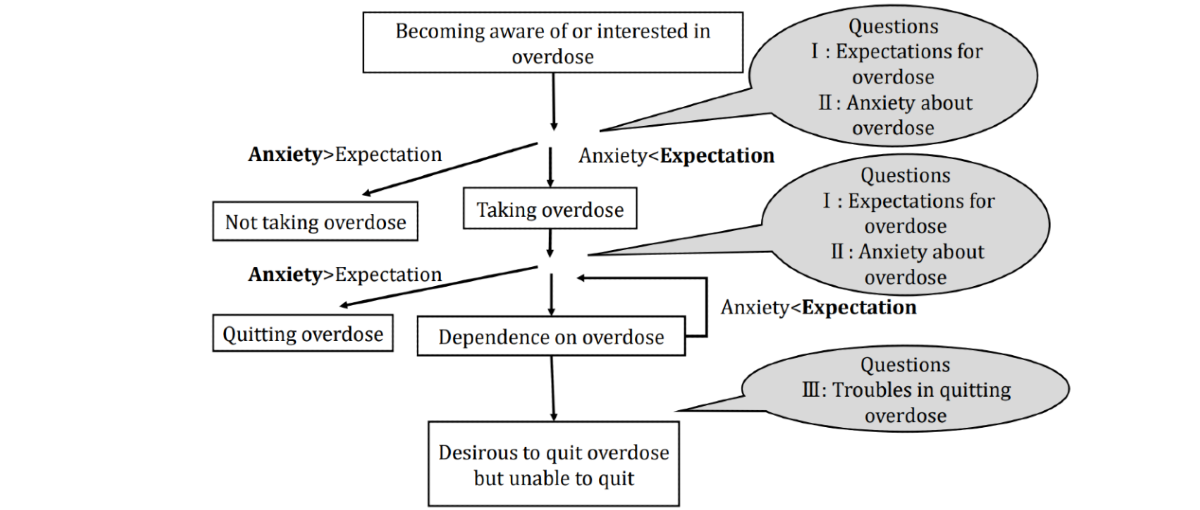
Conceptual diagram of results.

First, people who were aware of and interested in overdose asked questions about *theme 1: expectations for overdose*, such as the methods and benefits that can be obtained from overdose, and *theme 2: anxieties about overdose*, such as concerns about the effects of overdose on the body. When expectations outweighed concerns about overdose, overdose was taken. People who had experienced overdose asked questions about how they could get more benefits (*theme 1: expectations for overdose*) and about the pain that actually happened to them because of overdose (*theme 2: anxiety about overdose*). People at a stage where their expectations for overdose were in excess and they were dependent on overdose further asked about their expectations for and concerns about overdose. During the dependence on overdose, a flow of questions such as asking how to stop overdose at the stage where anxiety exceeds expectation (*theme 3: troubles in quitting overdose*) was made clear. A conceptual diagram of the results is shown in Figure 3.

## Discussion

### Principal Findings

#### Overview

In this research, we attempted to identify questions that people who potentially abuse OTC drugs may have by analyzing the questions about OTC drug abuse posted on the Q&A website, Yahoo! Chiebukuro. We confirmed that Yahoo! Chiebukuro contained a lot of textual data on concerns and questions that could not be obtained from medical institutions or public surveys, as well as those on background information leading to OTC drug abuse. As a result, 3 themes were generated: expectations for overdose, anxieties about overdose, and troubles in quitting overdose. On the basis of the results obtained from this study, we discuss the following 3 points.

#### The Conflict Between Expectations and Anxieties for Abusing OTC Drugs

In the questions posted on CGM, there were expectations and anxieties for OTC drug abuse, as reflected in *theme 1: expectations for overdose* and *theme 2: anxieties about overdose*. Although euphoria was the most common expected effect, there were also descriptions of seeking effects such as hallucinations, hematemesis, and breakdown. Owing to the difference in the effects expected from OTC drug abuse, there are 2 major types of abuse cases: the one for pleasure and the other for self-harm or suicide.

People who abuse OTC drugs often experience mental distress in various settings such as at home, school, and work [4]. In addition, it is said to be clinically apparent that people are trying to get by with OTC drug abuse because they are unable to discuss their situation with others [4]. These are common backgrounds for both types of abovementioned abuse cases [4,34]. In many cases, there is a desire to become free from emotional pain by obtaining pleasure or by hurting oneself. However, they do not wish to experience serious physical symptoms. They are presumed to have posted their questions while conflicted between expectation and anxiety. Furthermore, *theme 3: troubles in quitting overdose* indicates a strong dependence of abusing OTC drugs. We assume that once people become dependent on OTC drug abuse, they have difficulties in stopping it on their own and need professional assistance. However, the general public is rarely aware of the dangers of dependence on OTC drugs, and in many cases, they do not know how to seek assistance to break free from their dependence [26]. The content of theme 3 is also related to theme 2, as it includes questions about the dependence of overdose in *theme 2: anxieties about overdose*. Furthermore, *theme 3: troubles in quitting overdose* has to do with concerns not only for people who potentially abuse OTC drugs but also for those who visit medical institutions.

#### The Role of CGM Among People Who Abuse OTC Drugs

The study revealed that people who abuse OTC drugs were posting questions about OTC drug abuse on Q&A websites and that the number of such postings was increasing. Many types of CGM, including Yahoo! Chiebukuro, which is the subject of this research, can produce information anonymously. In addition, CGM has the characteristic that it does not function if the user’s information is not transmitted [23]. Q&A websites, which are part of CGM, are considered to be recognized and used by certain people who are abusing and trying to abuse OTC drugs as sources for quickly obtaining the information they seek without disclosing their identity [35]. This assumption is based on the recognition that OTC drug abuse is difficult to discuss face-to-face. Some of the questions indicated that people who abuse drug were anxious that other people around them would know that they were abusing drugs. Therefore, they seemed to recognize that their abuse was not something that can be openly discussed with their acquaintances.

In 2 of the themes of this study, *theme 2: anxieties about overdose* and *theme 3: troubles in quitting overdose*, there were several questions that should be discussed directly with the medical institution. However, the analysis confirmed that people who abuse drugs were anxious about even telling their medical institutions about their abuse and that they did not necessarily want a situation where they cannot abuse.

Thus, it is presumed that people who abuse OTC drugs may feel lonely and seek information about OTC drug abuse on various types of CGM, including Q&A websites. In addition, reliable information for those who abuse OTC drugs may not be available. We believe that information on OTC drug abuse needs to be developed so that people who potentially abuse OTC drugs do not fall into a state of serious dependence and can stop abusing to get rid of a state of dependence [36]. Given the current lack of reliable information for people who abuse OTC drugs, first of all, it is necessary to develop, in consultation with experts, information on how to seek help when they want to quit abuse but are unable to quit [26]. Rather than placing restrictions on Q&A websites, we believe that there needs to be a system that allows users to obtain reliable information on how to seek help when they want to stop but cannot, etc, at the time they search for information on OTC drug abuse through search engines.

#### Support by Pharmacies and Drugstores

Among the results of theme 1 was a question asked about how to buy medicine. People who abuse OTC drugs purchase their medications primarily at pharmacies and drugstores and over the internet, where they can purchase medications with no restrictions. In Japan, the sales volume of drugs that may be abused is limited. When selling drugs of potential abuse, several checks are set for the purchaser [37]. First, the name and age of the purchaser should be verified if he or she is young. Second, it should be ensured that similar drugs are not purchased at other pharmacies and drugstores. Third, the reason for purchase should be ascertained in the case where the purchaser intends to buy drugs in excess of the quantity deemed necessary for proper use. It is also stipulated that other items necessary to check that the purchase is for proper use should be verified. However, a survey showed that in approximately half of in-store sales, customers were able to make a purchase without being asked any questions, once they notified the pharmacy and drugstore that they were buying multiple units of a drug that could be abused or otherwise misused [25]. In addition, class-2 OTC drugs, which include drugs with a risk of abuse, have been sold on the internet since 2014 [38]. When customers made purchases on the internet after informing the sales company that they were purchasing multiple units of a drug that could be abused, more than half of them were able to make a purchase without being asked any questions [25]. These facts suggest that not enough research is being conducted to prevent OTC drug abuse. As pharmacists and registered sellers who sell pharmaceuticals are in a position to recognize the presence of people who abuse OTC drugs at an early stage, they should be able to play an important role as gatekeepers [11]. Therefore, support from pharmacies and drugstores needs to be instilled in society to reduce opportunities for OTC drug abuse.

### Limitations

As the extracted data in this study were limited to Yahoo! Chiebukuro, it cannot be said that the same results could be obtained from all types of CGM. In addition, because text data were searched and extracted only for specific words in this study, comprehensiveness was limited. In addition, it is difficult to determine whether the number of questions is a reasonable number, because previous studies in Japan are limited and the actual number of people who potentially abuse OTC drugs cannot be predicted. Finally, the possibility that the service provider had filtered out inappropriate content cannot be excluded. If this was the case, the results might have been underestimated.

### Conclusions

On CGM, questions were posted regarding expectations for and anxieties about abusing OTC drugs. Questions were also posted asking for advice on quitting repeated abuse and dependence. The fact that CGM was used to exchange information about OTC drug abuse suggests that it is necessary to produce and disseminate information on OTC drug abuse.

## Abbreviations

CGM: consumer-generated media
FY: fiscal year
OTC: over-the-counter
Q&A: question and answer
